# Attention Mechanisms and Their Applications to Complex Systems

**DOI:** 10.3390/e23030283

**Published:** 2021-02-26

**Authors:** Adrián Hernández, José M. Amigó

**Affiliations:** Centro de Investigación Operativa, Universidad Miguel Hernández, Av. de la Universidad s/n, 03202 Elche, Spain; adrian.hernandez@goumh.umh.es

**Keywords:** attention, deep learning, complex and dynamical systems, self-attention, neural networks, sequential reasoning

## Abstract

Deep learning models and graphics processing units have completely transformed the field of machine learning. Recurrent neural networks and long short-term memories have been successfully used to model and predict complex systems. However, these classic models do not perform sequential reasoning, a process that guides a task based on perception and memory. In recent years, attention mechanisms have emerged as a promising solution to these problems. In this review, we describe the key aspects of attention mechanisms and some relevant attention techniques and point out why they are a remarkable advance in machine learning. Then, we illustrate some important applications of these techniques in the modeling of complex systems.

## 1. Introduction

The combination of deep neural networks and the computational capabilities of Graphics Processing Units (GPUs) [1] has brought a breakthrough to the field of machine learning, improving the performance of several tasks such as image recognition, machine translation, language modelling, time series prediction, etc. [2,3,4,5].

Recurrent neural networks (RNNs) and long short-term memories (LSTMs), which were specially designed for sequence modelling [6,7,8,9], and convolutional neural networks (CNNs) to a lesser extent, have been successfully used to model, analyze and predict complex systems. Indeed, they are able to capture temporal dependencies and nontrivial relationships in complex systems, specifically in the sequential data generated by them. By complex systems we mean, generally speaking, systems that evolve over time in a possibly more general setting than that of dynamic systems.

However, these classic deep learning models do not perform sequential reasoning [10], a process that is based on perception with attention. In the brain, attention mechanisms allow to focus on one part of the input or memory (image, text, etc) while giving less attention to others, thus guiding the process of reasoning.

Attention mechanisms have provided and will provide a paradigm shift in machine learning [11,12]. These mechanisms allow a model to focus only on a set of elements and to decompose a problem into a sequence of attention based reasoning tasks [13]. Moreover, they can be applied to model complex systems in a flexible and promising way. When it comes to their application, information processing in the system and internal structure are crucial.

Here, as shown in Table 1, we describe the evolution of machine learning techniques and demonstrate how attention mechanisms, in combination with classic models, allow modeling certain important characteristics of complex systems, e.g., sequential reasoning, integration of different parts and long term dependencies.

In this paper, which is aimed at researchers with prior knowledge of deep learning, we review recent progress in attention mechanisms. We focus on differentiable attention, in which the attention weights are learned together with the rest of the model parameters. In Section 2, we present a general overview of the use of deep learning in modeling dynamical systems and, more generally, complex systems. We also elaborate on the need for attention mechanisms. In Section 3, we present the key aspects, the advantages and the main modes of operation of attention (Section 3.1). Then we describe some important attention techniques such as attention in seq2seq models (Section 3.2), as well as self-attention and memory networks (Section 3.3), emphasizing why they represent significant progress in machine learning. Finally, in Section 4.1, Section 4.2, Section 4.3 and Section 4.4 we illustrate some interesting uses of theses techniques to model complex systems and in Section 5 we discuss these techniques. For the convenience of the reader, all abbreviations in this paper are listed last.

## 2. Traditional Deep Learning and the Need for Attention

In recent years, we have seen major advances in the field of artificial intelligence and machine learning. The combination of deep neural networks with the computational capabilities of Graphics Processing Units (GPUs) [1] has improved the performance of several tasks such as image recognition, machine translation, language modelling, time series prediction, game playing and more [2,3,4,5]. Deep learning models have evolved to take into account the computational structure of the problem to be resolved.

**Figure 1:**
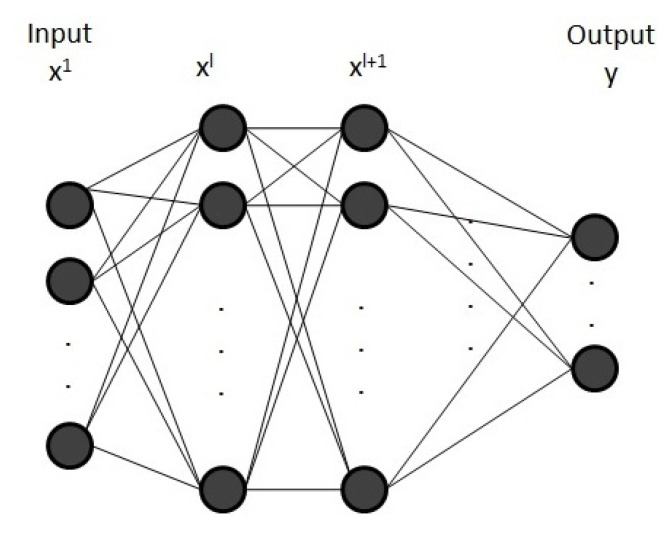
Multilayer neural network.

In a feedforward neural network (FNN) composed of multiple layers, the output (without the bias term) at layer *l*, see Figure 1, is defined as

(1) $$x^{l+1}=f\left(W^{l}x^{l}\right),$$

$W^{l}$ being the weight matrix at layer *l*. *f* is the activation function and $x^{l+1}$, the output vector at layer *l* and the input vector at layer l+1. The weight matrices for the different layers are the parameters of the model.

Learning is the mechanism by which the parameters of a neural network are adapted to the environment in the training process. This is an optimization problem that has been addressed using gradient-based methods, in which given a cost function $f:R^{n}\to R$, the algorithm finds local minima $w^{*}=\;{arg min}_{w}f\left(w\right)$ by updating each layer parameter $w_{ij}$ with the rule $w_{ij}:=w_{ij}-\eta \nabla_{w_{ij}}f\left(w\right)$, where η>0 is the learning rate.

Therefore, a deep learning model consists of the forward pass, in which the computational graph with the multiple layers is built, and the backward pass, in which the gradients are calculated and the parameters are updated. Then, all the functions of the parameters used in the model must be differentiable.

**Figure 2:**
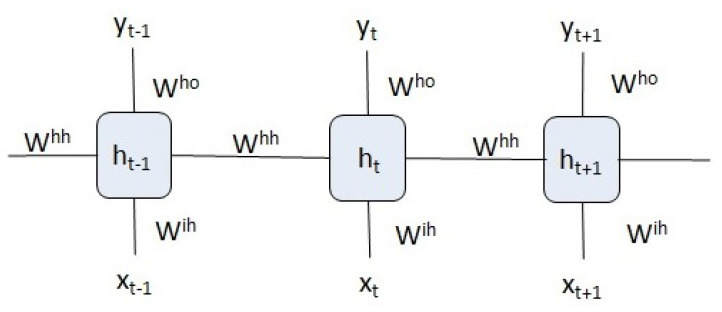
Temporal structure of a recurrent neural network.

RNNs (see Figure 2) are a basic component of modern deep learning architectures, especially of encoder–decoder networks. The following equations define the time evolution of an RNN:

(2) $$h_{t}=f^{h}\left(W^{ih}x_{t}+W^{hh}h_{t-1}\right),$$

(3) $$y_{t}=f^{o}\left(W^{ho}h_{t}\right),$$

where $W^{ih}$, $W^{hh}$ and $W^{ho}$ are weight matrices. $f^{h}$ and $f^{o}$ are the hidden and output activation functions while $x_{t}$, $h_{t}$ and $y_{t}$ are the network input, hidden state and output.

LSTMs [14] are an evolution of RNNs in that they feature an RNN structure with gated units, i.e., regulators. Specifically, LSTMs are composed of a memory cell, an input gate, an output gate and a forget gate, and allow gradients to flow unchanged. The memory cell remembers values over arbitrary time intervals and the three gates regulate the flow of information into and out of the cell.

Here we refer to systems that contain a very large number of variables interacting with each other in non-trivial ways as complex systems [15]. Their behaviour is intrinsically difficult to model due to the dependencies and interactions between their parts and they have emergence properties arising from those interactions such as adaptation, evolution, learning, etc. In Section 4, we describe the use of attention mechanisms to model the sequential data generated by complex systems.

Dynamical systems are a special class of complex systems. At any given time, a dynamical system has a state that can be represented by a point in a state space (manifold). The evolution equations of the dynamical system describes what future states follow from the current state. This process can be deterministic, if its entire future is uniquely determined by its current state, or non-deterministic otherwise [16] (e.g., a random dynamical system [17]). Furthermore, it can be a continuous-time process, represented by differential equations or a discrete-time process, represented by difference equations or maps. Thus,

(4) $$h_{t}=f\left(h_{t-1};\theta \right)$$

for autonomous discrete-time deterministic dynamical systems with parameters θ, and

(5) $$h_{t}=f\left(h_{t-1},x_{t};\theta \right)$$

for non-autonomous discrete-time deterministic dynamical systems driven by an external input $x_{t}$. Dynamical systems with multiple time lags can be rewritten as a higher dimensional dynamical system with time lag 1.

A key aspect in modelling dynamical systems is, of course, temporal dependence. Traditionally, there have been two ways to implement it in the neural network paradigm [18]:

1. Classic feedforward neural networks with time delayed states in the inputs but perhaps with an unnecessary increase in the number of parameters.
2. RNNs since, as shown in Equations (2) and (3), they have a temporal recurrence that make them appropriate for modelling discrete dynamical systems of the form given in Equations (4) and (5). As said in the Introduction, RNNs were precisely designed for sequence modelling. [6].

Therefore, RNNs seem the ideal candidates to model, analyze and predict dynamical systems and more generally complex systems. Theoretically, the temporal recurrence of RNNs allows to model and identify dynamical systems described with equations with any temporal dependence.

To learn chaotic dynamics, recurrent radial basis function (RBF) networks [19] and evolutionary algorithms that generate RNNs have been proposed [20]. “Nonlinear Autoregressive model with exogenous input” (NARX) [21] and boosted RNNs [22] have been applied to predict chaotic time series.

LSTMs have also succeeded in various applications to complex systems such as model identification and time series prediction [7,8,9]. Another remarkable application of the LSTM is machine translation [3,23].

Although the classic models above work well, they have limitations that make it difficult to perform sequential reasoning and achieve more general intelligence [10,24]. Among these limitations, we highlight the following:

1. Classic models only perform perception, representing a mapping between inputs and outputs.
2. Classic models follow a hybrid model where synaptic weights perform both processing and memory tasks but do not have an explicit external memory.
3. Classic models do not carry out sequential reasoning. This essential process is based on perception and memory through attention and guides the steps of the machine learning model in a conscious and interpretable way.

In the next section, we present attention mechanisms as an important step to address these limitations.

## 3. Attention Mechanisms

### 3.1. Differentiable Attention

As explained in Section 2, classic deep learning models do not perform sequential reasoning, a process that is based on attention.

In the brain, reasoning is the process of establishing and verifying facts combining attention with new or existing information. The role of the attention mechanisms is to focus on one part of the input or memory (image, text, etc), thus guiding the process of reasoning.

As described in [25], there are several classes of attention in neuroscience: attention as a level of alertness, attention over sensory inputs, attention to select and execute tasks and attention for memory encoding and retrieval. In [26], the authors modeled the interaction between top-down attention and bottom-up stimulus contrast effects and found that external attention inputs bias neurons to move to different parts of their nonlinear activation functions. Insects have been a source of inspiration in intelligence and attention mechanisms. In [27], a multiclass support vector machine with inhibition is inspired by the brain structure of insects. In [28], a multi-layer spiking neural network is presented that models the Mushroom Bodies and their interactions to other key elements of the insect brain, the Central Complex and the Lateral Horns.

Analogously, a learning problem in machine learning can be decomposed into a sequence of tasks, where in each task it is necessary to focus on one part of an input (or transformed input) or a memory. Once again, neural information processing in the brain, in which several layers interact with each other [29], has been a source of inspiration for machine learning.

Generally formulated, attention in machine learning is a sequential process in which a learning task is guided by a set of elements of the input source (or memory). This is achieved by integrating the attention value into the task.

Attention mechanisms have provided and will provide a paradigm shift in machine learning. Specifically, this change is from traditional large-scale vector transformations to more conscious processes (i.e., that focus only on a set of elements), e.g., decomposing a problem into a sequence of attention based reasoning tasks [13,30,31,32,33,34].

As stated in Section 2, to integrate a component into a deep learning model that learns using gradient descent, all the functions of the parameters in the component must be differentiable. One way to make attention mechanisms differentiable is to formulate them as a convex combination of the input or memory. In this case, all the steps are differentiable and can be learned, and the combination weights must add up to one (forcing them to focus on some parts more than others). In this way, the mechanisms learn which parts it needs to focus on.

**Figure 3:**
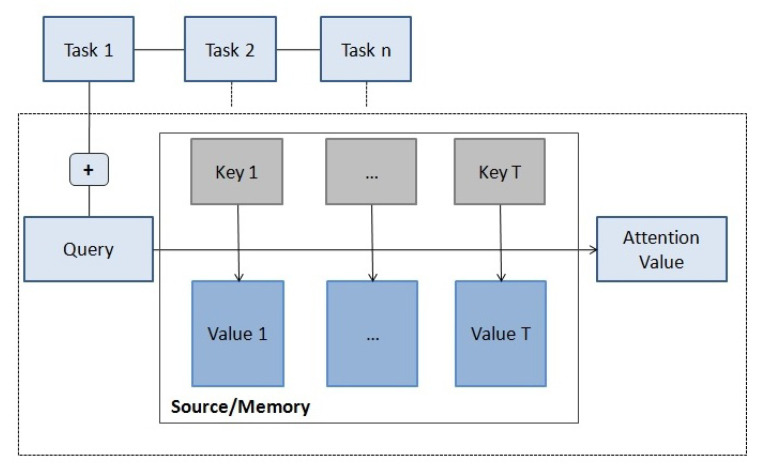
Attention diagram. Attention as a sequential process of reasoning in which the task (query) is guided by a set of elements (values) of the source (or memory).

As in [11], this convex combination, shown in Figure 3, is described as mapping a query and a set of key-value pairs to an output:

(6) $$att\left(q,s\right)=\sum_{i=1}^{T}\alpha_{i}\left(q,k_{i}\right)V_{i},$$

where, as seen in Figure 3, $k_{i}$ and $V_{i}$ are the key and the value vectors from the source/memory s, and q is the query vector (task). $\alpha_{i}\left(q,k_{i}\right)$ is the similarity function between the query and the corresponding key and is calculated by applying the softmax function,

(7) $$Softmax\left(z_{i}\right)=\frac{\exp(z_{i})}{\sum_{i^{\prime }}\exp\left(z_{i^{\prime }}\right)},$$

to the score function $score(q,k_{i})$:

(8) $$\alpha_{i}=\frac{\exp(score\left(q,k_{i}\right))}{\sum_{i^{\prime }=1}^{T}\exp\left(score\left(q,k_{i^{\prime }}\right)\right)}.$$

The score function can be computed using a feedforward neural network:

(9) $$score\left(q,k_{i}\right)=Z_{a}\tanh\left(W_{a}\left[q,k_{i}\right]\right)),$$

as proposed in [35], where $Z_{a}$ and $W_{a}$ are matrices to be jointly learned with the rest of the model and $\left[q,k_{i}\right]$ is a linear function or concatenation of q and $k_{i}$. Furthermore, in [36] the authors use a cosine similarity measure for content-based attention, namely,

(10) $$score\left(q,k_{i}\right)=\cos\left(\left(q,k_{i}\right)\right),$$

where $\left(\left(q,k_{i}\right)\right)$ denotes the angle between q and $k_{i}$.

Then, attention can be seen as a sequential process of reasoning in which the task (query) is guided by a set of elements of the input source (or memory) using attention.

The attention process can focus on:

1. Temporal dimensions, e.g., different time steps of a sequence.
2. Spatial dimensions, e.g., different regions of an image.
3. Different elements of a memory.
4. Different features or dimensions of an input vector, etc.

Depending on where the process is initiated, we have:

1. Top-down attention, initiated by the current task.
2. Bottom-up, initiated spontaneously by the source or memory.

To apply the attention mechanism, it is necessary to break down the learning process into a sequence of attention-guided tasks.

Then, due to its flexibility, an attention mechanism can be added in multiple ways to any deep learning architecture that models a complex system. In Section 4, we illustrate this flexibility as follows:

1. Through a conventional attention (the query is different from the key and the value) in Section 4.2, with the encoder selecting input features and the decoder selecting time steps.
2. Through a memory network in which a memory of historical data guides the current prediction task in Section 4.3.
3. Through self-attention (the keys, values and queries come from the same source) in Section 4.4. Here, to encode a vector of the input sequence, self-attention allows the model to focus in a direct way on other vectors in the sequence.

### 3.2. Attention in seq2seq Models

An encoder–decoder model maps an input sequence to a target one with both sequences of arbitrary length [3]. They have applications ranging from machine translation to time series prediction.

**Figure 4:**
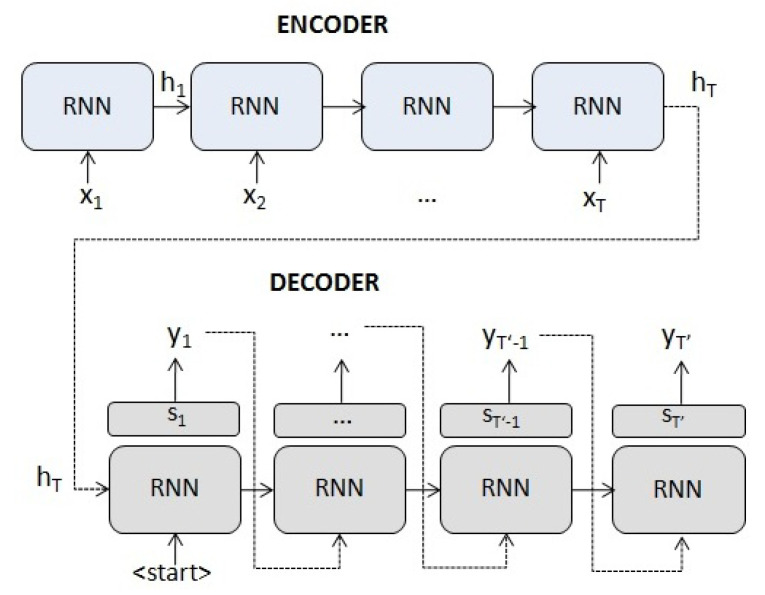
An encoder–decoder network.

More specifically, this mechanism uses an RNN (or any of its variants such as an LSTM or a GRU, Gated Recurrent Unit) to map the input sequence to a fixed-length vector, and another or any of its variants (RNN) to decode the target sequence from that vector (see Figure 4). Such a seq2seq model typically features an architecture composed of:

1. An encoder which, given an input sequence $X=(x_{1},x_{2},\ldots ,x_{T})$ with $x_{t}\in R^{n}$, maps $x_{t}$ to
  (11) $$h_{t}=f_{1}\left(h_{t-1},x_{t}\right),$$
  where $h_{t}\in R^{m}$ is the hidden state of the encoder at time *t*, *m* is the size of the hidden state and $f_{1}$ is an or any of its variants (RNN).
2. A decoder, where $s_{t}$ is the hidden state and whose initial state $s_{0}$ is initialized with the last hidden state of the encoder $h_{T}$. It generates the output sequence $Y=(y_{1},y_{2},\ldots ,y_{T^{\prime }})$, $y_{t}\in R^{o}$ (the dimension *o* depending on the task), where
  (12) $$y_{t}=f_{2}\left(s_{t-1},y_{t-1}\right),$$
  and $f_{2}$ is an or any of its variants (RNN) with an additional layer depending on the task (e.g., a linear layer for series prediction or a softmax layer for translation).

Because the encoder compresses all the information of the input sequence in a fixed-length vector (the final hidden state $h_{T}$), the decoder possibly does not take into account the first elements of the input sequence. The use of this fixed-length vector is a limitation to improve the performance of the encoder–decoder networks. Moreover, the performance of encoder–decoder networks degrades rapidly as the length of the input sequence increases [37]. This occurs in applications such as machine translation and time series prediction, where it is necessary to model long time dependencies.

The key to solve this problem is to use an attention mechanism to guide the decoding task. In [35], an extension of the basic encoder–decoder architecture was proposed by allowing the model to automatically search and learn which parts of a source sequence are relevant to predict the target element. Instead of encoding the input sequence in a fixed-length vector, it generates a sequence of vectors, choosing the most appropriate subset of these vectors during the decoding process.

Equipped with the attention mechanism, the encoder is a bidirectional RNN [38] with a forward hidden state $\vec{h_{i}}=f_{1}\left({\vec{h}}_{i-1},x_{i}\right)$ and a backward one $\overset{\leftarrow }{h_{i}}=f_{1}\left({\overset{\leftarrow }{h}}_{i+1},x_{i}\right)$. The encoder state is represented as a simple concatenation of the two states,

(13) $$h_{i}=\left[\vec{h_{i}};\overset{\leftarrow }{h_{i}}\right],$$

with i=1,…,T. The encoder state includes both the preceding and following elements of the sequence, thus capturing information from neighbouring inputs.

The decoder has an output

(14) $$y_{t}=f_{2}\left(s_{t-1},y_{t-1},c_{t}\right)$$

for $t=1,\ldots ,T^{\prime }$. $f_{2}$ is an RNN with an additional layer depending on the task (e.g., a linear layer for series prediction or a softmax layer for translation), and the input is a concatenation of $y_{t-1}$ with the context vector $c_{t}$, which is a sum of hidden states of the input sequence weighted by alignment scores:

(15) $$c_{t}=\sum_{i=1}^{T}\alpha_{ti}h_{i}.$$

Similar to Equation (8), the weight $\alpha_{ti}$ of each state $h_{i}$ is calculated by

(16) $$\alpha_{ti}=\frac{\exp(score\left(s_{t-1},h_{i}\right))}{\sum_{i^{\prime }=1}^{T}\exp\left(score\left(s_{t-1},h_{i^{\prime }}\right)\right)}.$$

**Figure 5:**
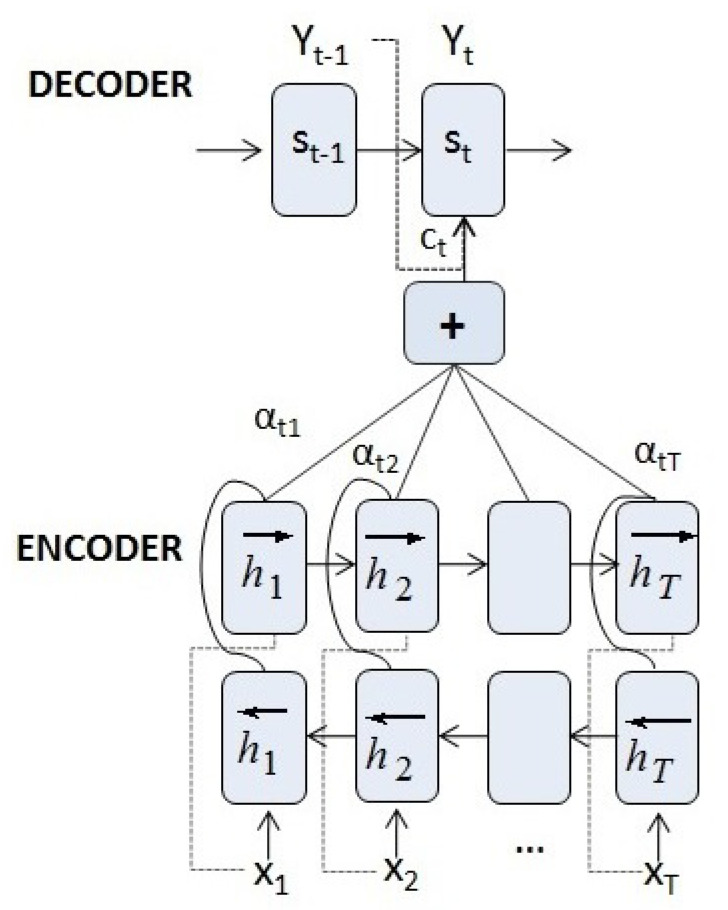
An encoder–decoder network with attention.

In this attention mechanism, the query is the state $s_{t-1}$ and the key and the value are the hidden states $h_{i}$. The score measures how well the input at position *i* and the output at position *t* match. $\alpha_{ti}$ are the weights that implement the attention mechanism, defining how much of each input hidden state should be considered when deciding the next state $s_{t}$ and generating the output $y_{t}$ (see Figure 5).

As we have described previously, the score function can be parametrized using different alignment models such as feedforward networks and the cosine similarity.

**Figure 6:**
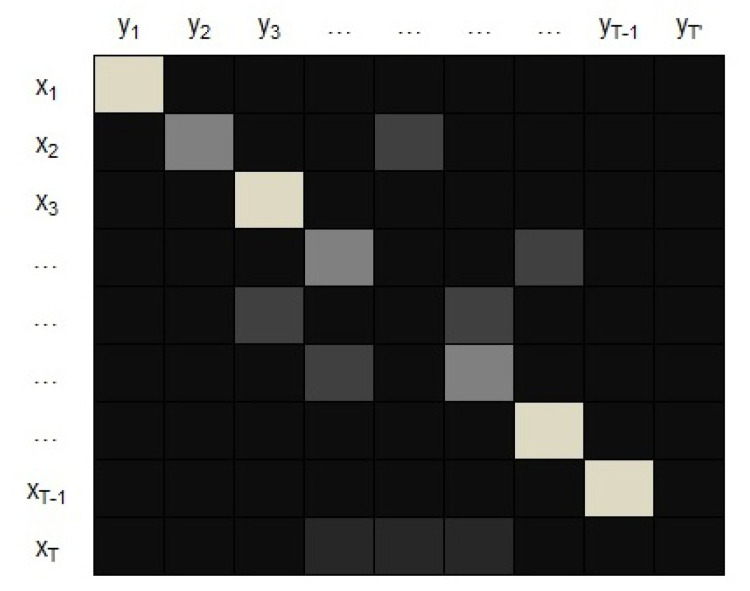
A matrix of alignment scores. It represents how much of each input state should be considered when deciding the next state and generating the output.

An example of a matrix of alignment scores is shown in Figure 6. This matrix provides interpretability to the model since it allows to know which part (time-step) of the input is more important to the output.

The attention mechanism then transforms an encoder–decoder sequential model into a non-sequential model in which the attention mechanism guides the decoding task based on the encoded states.

### 3.3. Self-Attention and Memory Networks

A variant of the attention mechanism is self-attention, in which the attention component relates different positions of a single sequence in order to compute a representation of the sequence. In this way, the keys, values and queries come from the same source. The mechanism can connect distant elements of the sequence more directly than using RNNs [12].

Similar to the description given in [11], for an input sequence $X=(x_{1},x_{2},\ldots ,x_{T})$, the self-attention process can be implemented by the following steps:

1. For each of the input vectors, create a query $Q_{t}$, a key $K_{t}$ and a value vector $V_{t}$ by multiplying the input vector $x_{t}$ by three matrices that are trained during the learning process, $W_{i}^{Q}\in R^{d\times d_{k}},W_{i}^{K}\in R^{d\times d_{k}}$ and $W_{i}^{V}\in R^{d\times d_{v}}$.
2. For each query vector $Q_{t}$, the self-attention value is computed by mapping the query and all the key-values to an output, $Attention\left(Q_{t},K,V\right)=\sum_{j=1}^{T}\alpha_{j}\left(Q_{t},K_{j}\right)V_{j}$, where
  (17) $$\alpha_{j}\left(Q_{t},K_{j}\right)=softmax\left(\frac{Q_{t}K_{j}^{T}}{\sqrt{d_{k}}}\right)$$
3. This self-attention process is performed *h* times in what is called multi-headed attention. Each time, the input vectors are projected into a different query, key and value vector using different matrices $W_{i}^{Q},W_{i}^{K}$ and $W_{i}^{V}$ for i=1,…,h. On each of these projected queries, keys and values, the attention function is performed in parallel, producing $d_{v}$ dimensional output values, that are concatenated and once again projected to the final values. This multi-headed attention process allows the model to focus on different positions from different representation subspaces.

**Figure 7:**
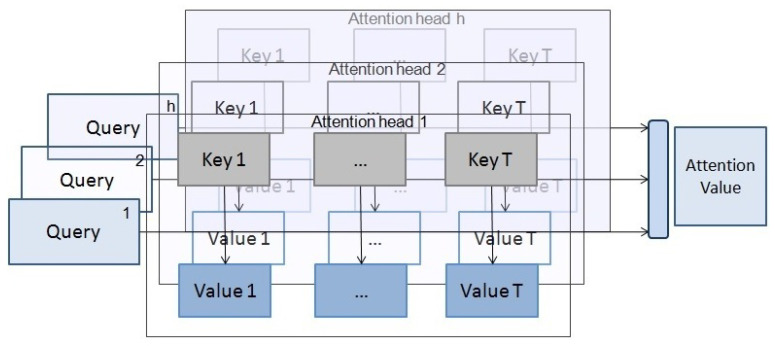
Multi-headed attention. Self-attention process performed in parallel *h* times in different subspaces. The output values are concatenated and projected to a final value.

When the model is processing a vector of the input sequence, single self-attention allows the model to focus on other vectors in the sequence to get a better representation of this vector. With multi-headed self-attention (see Figure 7), each attention head is focusing on a different set of vectors when processing the vector.

The transformer [11], a network architecture based only on self-attention, is composed of an encoder and a decoder:

1. Encoder: Composed of a stack of six identical layers, each layer with a multi-head self-attention process and a position-wise fully connected feed-forward network. Around each of the sub-layers, a residual connections followed by layer normalization is employed.
2. Decoder: Is also composed of a stack of six identical layers (with self-attention and a feed-forward network) with an additional third sub-layer to perform attention over the output of the encoder (as in the seq2seq with attention). The self-attention sub-layer is modified to prevent a vector from attending to subsequent vectors in the sequence.

The transformer allows to replace CNNs and RNNs, improving machine translation tasks while using less training time. The transformer is also the basic component of GPT-3 (Generative Pre-Trained transformer-3), a pre-trained language model which achieves good performance in few-shot learning on many Natural Language Processing tasks without fine-tuning [39].

Another variant of attention are end-to-end memory networks [40], which are neural networks with a recurrent attention model over an external memory. The model, trained end-to-end, is described in more detail in Section 4.3 and outputs an answer based on a query and a set of inputs $x_{1},x_{2},\ldots ,x_{n}$ stored in a memory.

## 4. Attention Mechanisms in Complex Systems

### 4.1. Where and How to Apply Attention

In the previous sections we have described various attention mechanisms. These mechanisms allow a task to focus on a set of elements of an input sequence, an intermediate sequence or a memory source.

Due to its flexibility, an attention mechanism can be added to any deep learning model in multiple ways. Therefore, when applying it to model complex systems, it will be necessary to decide the following issues:

1. In which part of the model should be introduced?
2. What elements of the model will the attention mechanism relate?
3. What dimension (temporal, spatial, input dimension, etc.) is the mechanism going to focus on?
4. Will self-attention or conventional attention be used?
5. What elements will correspond to the query, the key and the value?

In the following sections we describe some illustrative cases of application of attention mechanisms to model complex systems. As we will see, how the information is processed in the system and how the different elements are related will be key when defining the aforementioned issues.

### 4.2. Attention in Different Phases of a Model

In a non-autonomous dynamical system, the current state is a transformation of the previous states and the current input, which contains *n* dimensions or features. More generally, the dependencies between time steps can be dynamic, i.e., time-changing. In such complex systems, attention mechanisms learn to focus on the most relevant parts of the system input or state.

A representative attention mechanism in this context implements a dual-stage attention, namely, an encoder with input features attention and a decoder with temporal attention, as pointed out in [41]. Next we describe this architecture, in which the first stage extracts the relevant input features and the second selects the relevant time steps of the model.

Let $X=(x_{1},x_{2},\ldots ,x_{T})$ with $x_{t}\in R^{n}$ be the input sequence. *T* is the length of the time interval and *n* the number of input features or dimensions. $x_{t}=\left(x_{t}^{1},x_{t}^{2},\ldots ,x_{t}^{n}\right)$ is the input at the time step *t* and $x^{k}=\left(x_{1}^{k},x_{2}^{k},\ldots ,x_{T}^{k}\right)$ is the *k* input feature series.

**Encoder with input attention**

Given an input sequence *X*, the encoder maps $u_{t}$ to

(18) $$h_{t}=f_{1}\left(h_{t-1},u_{t}\right),$$

where $h_{t}\in R^{m}$ is the hidden state of the encoder at time *t*, *m* is the size of the hidden state and $f_{1}$ is an or any of its variants (RNN). $x_{t}$ is replaced by $u_{t}$, which adaptively selects the relevant input features as follows:

(19) $$u_{t}=\left(\alpha_{t}^{1}x_{t}^{1},\alpha_{t}^{2}x_{t}^{2},\ldots ,\alpha_{t}^{n}x_{t}^{n}\right).$$

Here

(20) $$\alpha_{t}^{k}=\frac{\exp(score\left(h_{t-1},x^{k}\right))}{\sum_{i=1}^{n}\exp\left(score\left(h_{t-1},x^{i}\right)\right)},$$

is the attention weight measuring the importance of the *k* input feature at time *t*, where $x^{k}=\left(x_{1}^{k},x_{2}^{k},\ldots ,x_{T}^{k}\right)$ is the *k* input feature series and the score function can be computed using a feedforward neural network, a cosine similarity measure or other similarity functions.

**Figure 8:**
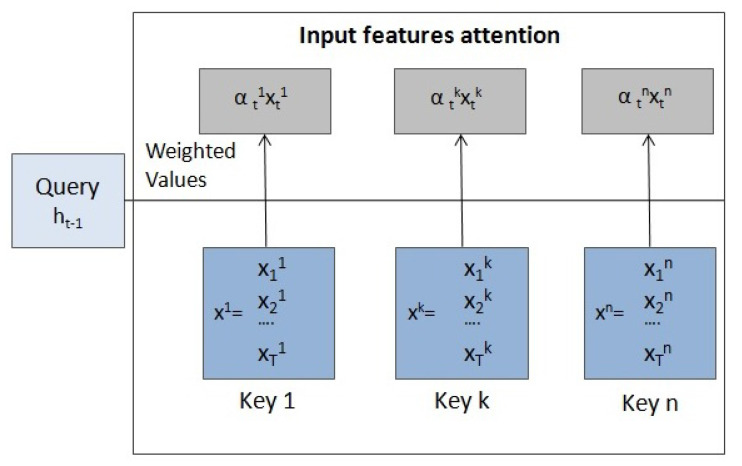
Diagram of the input features attention mechanism.

Therefore, this first attention stage extracts the relevant input features with the query, keys and values shown in Figure 8.

**Decoder with temporal attention**

Similar to the attention decoder described in Section 3.2, the decoder has an output

(21) $$y_{t}=f_{2}\left(s_{t-1},y_{t-1},c_{t}\right)$$

for $t=1,\ldots ,T^{\prime }$. $f_{2}$ is an or any of its variants (RNN) with an additional linear or softmax layer, and the input is a concatenation of $y_{t-1}$ with the context vector $c_{t}$, which is a sum of hidden states of the input sequence weighted by alignment scores:

(22) $$c_{t}=\sum_{i=1}^{T}\beta_{t}^{i}h_{i}.$$

The weight $\beta_{t}^{i}$ of each state $h_{i}$ is computed using the similarity function, $score(s_{t-1},h_{i})$, and applying a softmax function, as described in Section 3.2.

**Figure 9:**
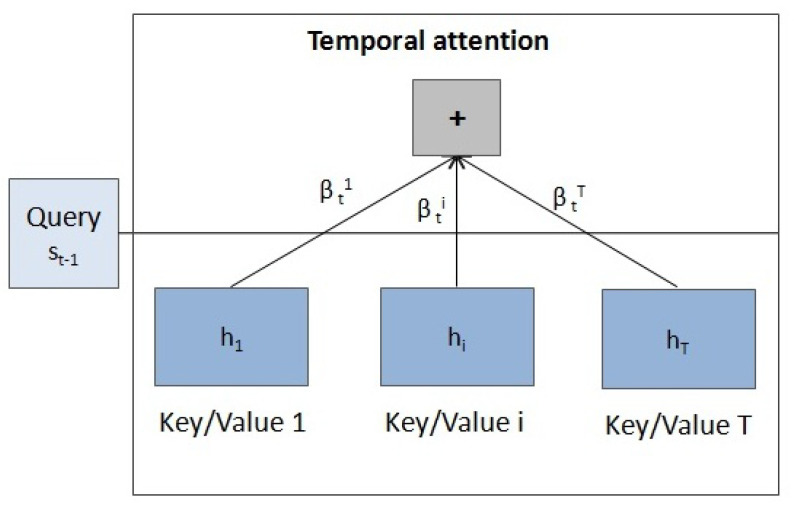
Diagram of the temporal attention mechanism.

This second attention stage selects the relevant time steps, as shown in Figure 9 with the corresponding query, keys and values.

**Further remarks**

In [41], the authors define this dual-stage attention RNN and show that the model outperforms a classical model in time series prediction.

A comparison is made between LSTMs and attention mechanisms for financial time series forecasting in [42]. It is shown that an LSTM with attention performs better than stand-alone LSTMs.

A temporal attention layer is used in [43] to select relevant information and to provide model interpretability, an essential feature to understand deep learning models. In [44], interpretability is further studied in detail, concluding that attention weights partially reflect the impact of the input elements on model prediction.

### 4.3. Memory Networks

Memory networks allow long-term or external dependencies in sequential data to be learned thanks to an external memory component. Instead of taking into account only the most recent states, memory networks also consider the entire list of states or the states of a memory.

Here we define one possible application of memory networks to complex systems, following an approach based on [40]. We are given a time series of historical data $n_{1},\ldots ,n_{T^{\prime }}$ with $n_{i}\in R^{n}$ and the input series $x_{1},\ldots ,x_{T}$ with $x_{t}\in R^{n}$ the current input, which is the query in the attenton mechanism.

The set $\left\{n_{i}\right\}$ are converted into memory vectors $\left\{m_{i}\right\}$ and output vectors $\left\{c_{i}\right\}$ of dimension *d*. The query $x_{t}$ is also transformed to obtain a internal state $u_{t}$ of dimension *d*. These transformations correspond to a linear transformation: $An_{i}=m_{i},Bn_{i}=c_{i},Cx_{t}=u_{t}$, where A,B,C are parameterizable matrices.

A match between $u_{t}$ and each memory vector $m_{i}$ is computed by taking the inner product followed by a softmax function:

(23) $$p_{t}^{i}=Softmax\left(u_{t}^{T}m_{i}\right).$$

The final vector from the memory, $o_{t}$, is a weighted sum over the transformed memory inputs $\left\{c_{i}\right\}$:

(24) $$o_{t}=\sum_{i}p_{t}^{i}c_{i}.$$

To generate the final prediction $y_{t}$, a linear layer is applied to the sum of the output vector $o_{t}$ and the transformed input $u_{t}$, and to the previous output $y_{t-1}$:

(25) $$y_{t}=f\left(W^{1}\left(o_{t}+u_{t}\right)+W^{2}y_{t-1}\right)$$

**Figure 10:**
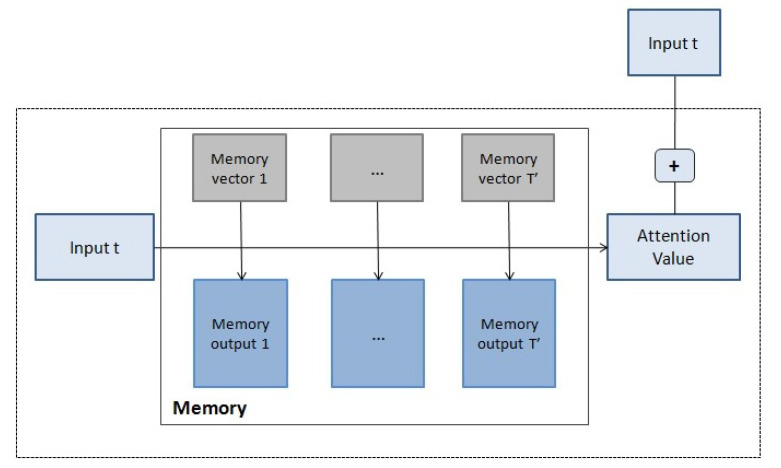
Basic diagram of a memory network. For each input, the attention mechanism integrates a weighted sum over the memory vectors.

A basic diagram of the model is shown in Figure 10. This model is differentiable end-to-end by learning the matrices (the final matrices $W^{1}$ and $W^{2}$, and the three transformation matrices A,B and *C*) to minimize the prediction error.

In [45], the authors propose a similar model based on memory networks with a memory component, three encoders and an autoregressive component for multivariate time-series forecasting. Compared to non-memory RNN models, their model is better at modeling and capturing long-term dependencies and, moreover, it is interpretable.

Differentiable Neural Computers (DNCs) [46] consist of a neural network that uses attention and can read from, and write to, an external memory. Taking advantage of these capabilities, an enhanced DNC for electroencephalogram (EEG) data analysis is proposed in [47]. By replacing the LSTM network controller with a recurrent convolutional network, the potential of DNCs in EEG signal processing is convincingly demonstrated.

### 4.4. Self-Attention

An important aspect to model complex systems is to capture the temporal dependence and the relationship between the parts that make up the system.

**Figure 11:**
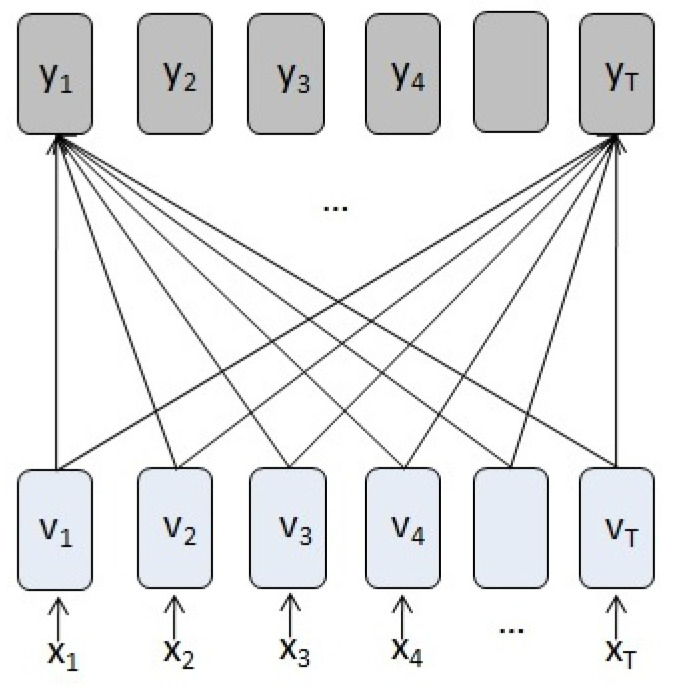
Self-attention graph. The self-attention component calculates how much each input vector contributes to form each output vector.

If we compare the computational graph of an see Figure 2 (RNN) with the graph of an attention module (Figure 11), we observe that even adding a memory unit (LSTM), the attention module relates each of the inputs in a more direct and symmetric way to form the output vector.

The distance, in number of edges in the graph, between an input and an output distant in time, is shorter and is the same for all input vectors in the self-attention module. However, this is at the cost of not prioritizing local interactions, which has a high computational cost for very long sequences.

The transformer, as we have pointed out, is composed of a stack of multi-headed self-attention components. With multi-headed attention, the input vectors are projected into a different query, key and value vector, performing the self-attention process *h* times. When processing a vector, each attention head is focusing on a different set of vectors from different representation subspaces.

These mentioned characteristics make self-attention and the transformer a promising building block in deep learning models for complex systems.

In [48], the authors propose a dual self-attention network for multivariate time (dynamic-period or non-periodic) series forecasting. In [49], the authors utilize attention models for clinical time-series modeling. They employ a masked self-attention mechanism and use positional encoding and dense interpolation for incorporating temporal order.

Further understanding of the transformer architecture is carried out in [50], where the authors show that the transformer architecture can be interpreted as a numerical Ordinary Differential Equation (ODE) solver for a convection-diffusion equation in a multi-particle dynamic system. They interpret how words (vectors) in a sentence are abstracted by passing through the layers of the transformer as approximating the movement of multiple particles in the space using the Lie–Trotter splitting scheme and the Euler’s method.

## 5. Discussion

After the success of recent years, one of the most important challenges that deep learning faces is to improve input-output models, adopting new primitives that provide reasoning, abstraction, search and memory capabilities.

Similar to what happens in the brain, attention mechanisms allow the reasoning or cognitive process to be guided in a flexible way. This improvement is important when modeling complex systems due to their temporal dependence and complex relationships.

As we have seen, attention mechanisms has the following benefits in modeling such systems:

1. By focusing on a subset of elements, it guides the reasoning or cognitive process.
2. These elements can be tensors (vectors) from the input layer, from the intermediate layer or be external to the model, e.g., a external memory.
3. It can focus on temporal dimensions (different time steps of a sequence), spatial dimensions (different regions of space) or different features of an input vector.
4. It can relate each of the input vectors in a more direct and symmetric way to form the output vector.

More specifically, as shown in Table 2, for each of the techniques and applications described, we discuss its application potential, characteristics and advantages.

1. *One stage conventional attention.* The attention mechanism allows guiding any complex system task such as modeling, prediction, identification, etc. To do this, it focuses on a set of elements from the input layer or from an intermediate layer. These elements can be temporal, spatial or feature dimensions. For example, to model a dynamical system with an input of dimension *n*, one can add an attention mechanism to focus and integrate the different input dimensions. The attention mechanism is combined, as we have seen, with an RNN or an LSTM and allows modeling long temporal dependencies. This technique, like the rest, adds complexity to the model. To calculate the attention weights between a task (query) of *T* elements and an attended region (key, value) of *T* elements, it is necessary to perform $T^{2}$ multiplications.
2. *Several stages conventional attention*. This case is similar to the previous, one-stage conventional attention but with several attention phases or stages. The attention mechanism is also combined with an RNN or an LSTM and allows modeling long temporal dependencies. As we have seen, the model can focus on a set of feature elements from the input layer and on a set of temporal steps from an intermediate layer. This enables multi-step reasoning. The downside is that more computational cost is added to the model with $T^{2}$ multiplications for each attention stage.
3. *Memory networks*. In memory networks, any complex system task such as modeling, prediction or identification is guided by an external memory. Then, memory networks allow long-term or external dependencies in sequential data to be learned thanks to an external memory component. Instead of taking into account only the most recent states, these networks consider the states of a memory or external data as well. Such is the case of time series prediction also based on an external source that can influence the series. To calculate the attention weights between a task (query) of *T* elements and an attended memory of $T^{\prime }$ elements, it is necessary to perform $TT^{\prime }$ multiplications.
4. *Self-attention*. In self-attention, the component relates different positions of a single sequence in order to compute a transformation of the sequence. The keys, values and queries come from the same source. It is a generalization of neural networks, since they perform a direct transformation of the input but the weights are dynamically calculated. The attention module relates each of the inputs in a more direct way to form the output vector but at the cost of not prioritizing local interactions. Their use case is general since they can replace neural networks, RNNs or even CNNs. To calculate the attention weights for a sequence of *T* elements it is necessary to perform $T^{2}$ multiplications.
5. *Combination of the above techniques*. It is interesting to combine several of the previous techniques but at the cost of increasing the complexity and adding the computational cost of each of the components. For example, the transformer, which can be used in a multitude of tasks such as sequence modeling, generative models, predictions, machine translation, multi-tasking, etc. The transformer combines self-attention with conventional attention. In the encoder, the transformer has a stack of self-attention blocks. The decoder also has self-attention blocks and an additional layer to perform attention over the output of the encoder.

However, despite the theoretical advantages and some achievements, further studies are needed to verify the benefits of the attention mechanisms over traditional networks in complex systems.

## Figures and Tables

**Table 1:** Classic deep learning and attention techniques described in this paper and their capabilities to model complex systems.

Techniques	Capabilities in Modeling Complex Systems
Classic models (RNNs, LSTMs *…*)	Are universal approximators, provide perception, temporal dependence and short memory
Seq2seq with attention	Integrates parts, models long term dependencies, guides a task by focusing on a set of elements (temporal, spatial, features *…*)
Memory networks	Integrate external data with the current task and provide an explicit external memory
Self-attention	Generalization of neural networks, relates input vectors in a more direct and symmetric way

**Table 2:** Characteristics of attention techniques in complex systems described in this paper.

Techniques	Operation	Use Cases	Costs	Applications
One-stage att.	Over the input or an	Integrate parts	Complexity	Modeling
	intermediate layer	Long term dependencies	$T^{2}$ operations	Prediction
	Temporal, spatial *…*			
Several stages	Over the input, over	Integrate several parts	Complexity	Modeling
	an intermediate layer	Long term dependencies	$T^{2}$ operations	Prediction
	Temporal, spatial *…*	Multi-step reasoning	each att. stage	Sequential reasoning
Memory networks	Over external data	Integrate a memory	Complexity	Modeling
	Temporal, spatial *…*		$TT^{\prime }$ operations	Reasoning over a memory
Self-attention	Relate elements	General	Complexity	Replace neural networks
	of the same sequence	Encode an input	Non local $T^{2}$ operations	
Combination	Combine the above elements	All of the above	Sum of the costs	All of the above

## Abbreviations

The following abbreviations are used in this manuscript:

DNC	Differentiable Neural Computers
CNN	Convolutional neural network
EEG	Electroencephalogram
FNN	Feed-forward neural network
GPT-3	Generative Pre-Trained transformer-3
GPU	Graphics Processing Units
LSTM	Long short-term memory
MDPI	Multidisciplinary Digital Publishing Institute
NARX	Nonlinear Autoregressive model with exogenous input
ODE	Ordinary Differential Equation
RBF	Recurrent radial basis function
RNN	Recurrent Neural Network
